# Organophosphates and Outdoor Air Concentrations

**DOI:** 10.1289/ehp.114-a338c

**Published:** 2006-06

**Authors:** Robert K.D. Peterson

**Affiliations:** Agricultural and Biological Risk Assessment, Department of Land Resources and Environmental Sciences, Montana State University, Bozeman, Montana, E-mail: bpeterson@montana.edu

Harnly et al. (2005) suggested that measured air concentrations of organophosphate insecticides may pose a particular concern for children’s health. However, when considering the current scientific weight of evidence, their conclusions cannot be supported for two reasons: first, they did not demonstrate a particular concern for children based on their results, and second, they cited incomplete and inappropriate literature to support the notion that recent toxicologic and epidemiologic studies indicate a health concern.

Harnly et al. (2005) did not conduct a risk assessment to demonstrate a concern for children. Rather, they detected pesticides in air concentrations in agricultural areas and then suggested there may be a concern for children because of recent toxicologic and epidemiologic studies. Their observed median exposures of all three active ingredients (chlorpyrifos, diazinon, and malathion) were all low and well within established regulatory limits. A risk assessment approach would have been quite useful. For chlorpyrifos, Harnly et al. (2005) detected a 20-day median concentration in air of 0.000033 mg/m^3^. A tier-1 risk assessment assuming an air concentration of chlorpyrifos at 0.000033 mg/m^3^, the mean body weight of a 1- to 2-year-old child of 12.3 kg, a child inhalation rate of 6.8 m^3^/day, and 24-hr outdoor respiration results in a chlorpyrifos inhalation exposure of 0.0000182 mg/kg/day. Margins of exposure (MOE) would be 5,495 [acute inhalation no observed adverse effect level (NOAEL) = 0.1 mg/kg/day], 27,473 (acute NOAEL = 0.5 mg/kg/day), and 1,648 (chronic NOAEL = 0.03 mg/kg/day). All MOEs are greater than the U.S. Environmental Protection Agency (EPA) target MOE of 1,000 for infants, children, and females 13–50 years of age (U.S. EPA 2002).

More problematic is that Harnly et al. (2005) stated that

Recent cellular, animal, and human evidence of toxicity, particularly in newborns, supports the public health concern indicated by initial risk estimates.

The authors did not provide a sufficiently thorough review of the literature relevant to risk assessment to support or refute their statement. In the case of chlorpyrifos, this statement cannot be supported by the available evidence. The principal problem with Harnly et al.’s approach is not unique to their article. Appropriate risk assessment requires appropriate data, and, as simple as this relationship sounds, it is often ignored. Harnly et al. (2005) cited toxicity and epidemiologic studies, but these particular studies are not appropriate for use in risk assessment. This problem has become so pervasive that Conolly et al. (1999) clarified the basic features of toxicology studies that are and are not appropriate for use in risk assessment.

Harnly et al. (2005) should not have included the findings of Qiao et al. (2002) because the high doses, subcutaneous route of administration, and carrier were inappropriate for toxicologic risk assessment (Conolly et al. 1999; Zhao et al. 2005). Indeed, Slotkin (2004), a coauthor of Qiao, has written that there is little academic interest in relevant routes of exposure or pharmacokinetics. He stated that

Practical issues that are critical to standardized testing are de-emphasized, such as pharmacokinetics/toxicokinetics, the matching of routes of exposure to those of humans in industrial, agricultural or domestic settings, or the development of biologically-based dose response models of established hazards. In that sense, the academic approach is entirely deficient in those attributes that are necessary components of the application of research findings to regulatory science.

Harnly et al. (2005) cited Eskenazi et al. (1999) as a source of concern for adverse consequences of organophosphate exposure. To be complete, Eskenazi et al. (2004) provide more information. They stated, “We failed to demonstrate an adverse relationship between fetal growth and any measure of *in utero* organophosphate pesticide exposure.” An association was found for a couple of variables and decreased gestational duration, but the conclusion was that these potential pesticide effects appear to have “little clinical impact at the population level.”

Finally, air concentrations have been shown to translate poorly into systemic exposure. Hore et al. (2005) showed that children in houses treated with chlorpyrifos had no detectable increase in urinary 3,5,6-trichloropyridinol (TCP), whereas median peak ambient air chlorpyrifos increased > 10-fold (median of 14 ng/m^3^ pretreatment, 196 ng/m^3^ on day of treatment). If a 10-fold increase in air chlorpyrifos does not cause a detectable increase in urinary TCP, then the 1-fold background air cannot be contributing measurably to the children’s background levels of urinary TCP.
